# Risk-Adapted Use of Adjuvant Systemic Therapy After Nephrectomy for Renal Cell Carcinoma: A Population-Based Study in the Post-approval Era

**DOI:** 10.1245/s10434-026-20341-9

**Published:** 2026-08-04

**Authors:** Maximilian Filzmayer, Michele Petix, Leonardo Quarta, Filippo Orlandi, Jordan A. Goyal, Luca Carmignani, Alberto Briganti, Salvatore Micali, Shahrokh F. Shariat, Mike Wenzel, Yasmine Heß-Busch, Clara Humke, Fred Saad, Felix K.-H. Chun, Pierre I. Karakiewicz

**Affiliations:** 1 Cancer Prognostics and Health Outcomes Unit, Division of Urology, University of Montreal Health Center, Montreal, Canada; 2 Department of Urology, University Hospital, Goethe University Frankfurt, Theodor-Stern-Kai 7, 60596 Frankfurt am Main, Germany; 3 Università degli Studi di Milano, Milan, Italy; 4 Department of Urology, IRCCS Policlinico San Donato, Milan, Italy; 5 Vita-Salute San Raffaele University, Milan, Italy; 6 Division of Experimental Oncology/Unit of Urology, Urological Research Institute, IRCCS San Raffaele Scientific Institute, Milan, Italy; 7 Department of Urology, AOU di Modena, University of Modena and Reggio Emilia, Modena, Italy; 8 Department of Urology, Comprehensive Cancer Center, Medical University of Vienna, Vienna, Austria; 9 Department of Urology, Weill Cornell Medical College, New York, NY USA; 10 Department of Urology, University of Texas Southwestern Medical Center, Dallas, TX USA; 11 Hourani Center of Applied Scientific Research, Al-Ahliyya Amman University, Amman, Jordan

**Keywords:** Kidney cancer, Renal cell carcinoma, Nephrectomy, High risk, Adjuvant systemic therapy

## Abstract

**Background:**

Real-world adjuvant systemic therapy (AST) use for eligible renal cell carcinoma (RCC) patients remains insufficiently characterized in the contemporary treatment era. In particular, it is unclear whether treatment allocation reflects established high-risk tumor characteristics.

**Methods:**

In the Surveillance, Epidemiology, and End Results (SEER) database, the study identified patients eligible for AST after nephrectomy (2018–2022). Temporal trends in AST use were assessed. Multivariable logistic regression models tested associations between histopathologic high-risk features (T4 stage, tumor grade 4, and N1–2 stage) and AST use.

**Results:**

Of 6,497 eligible patients, 1,163 (18%) received AST. Annual proportions of AST increased from 13% in 2018 to 36% in 2022. Higher AST use was independently predicted by T4 stage (33% vs. 18%; odds ratio [OR], 1.68; *p* = 0.006), tumor grade 4 (28% vs. 15%; OR, 1.94; *p* < 0.001), and N1–2 stage (36% vs. 17%; OR, 2.57; *p* < 0.001). Patients who had any of these three high-risk features were more likely to receive AST than those who did not (28% vs. 14%; OR, 2.27; *p* < 0.001). Moreover, a stepwise increase in AST use was observed with an increasing number of high-risk features (one: 26% [OR, 2.09; *p* < 0.001], two: 37% [OR, 3.56; *p* < 0.001], three: 40% [OR, 4.04; *p* = 0.014]).

**Conclusions:**

Only a minority of eligible RCC patients received AST after nephrectomy. However, the proportion of AST use increased over time. Use of AST was strongly associated with adverse tumor characteristics. A stratification according to the number of high-risk features showed a dose-response effect. Nevertheless, AST use remained limited even among patients at the highest risk.

Nephrectomy represents the standard of care for localized renal cell carcinoma (RCC).^1–3^ However, patients with adverse pathologic features remain at high risk for recurrence after nephrectomy, prompting the development and evaluation of adjuvant systemic therapies (ASTs).^4,5^ The approval of sunitinib in 2018 and pembrolizumab in 2022 established AST as a standard option for selected high-risk patients, based on randomized trial evidence and guideline recommendations.^1–3,6–9^

Prior population-based studies from the pre-approval era suggested that higher tumor stage, lymph node involvement, and high-grade histology were associated with increased off-label use of AST after nephrectomy.^10–12^ However, the population-based rates of AST use after regulatory approval are unknown, and it remains unclear whether treatment allocation reflects established histopathologic high-risk features, namely, T4 stage, tumor grade 4, and N1–2 stage.

We aimed to address these knowledge gaps and hypothesized that AST use increased over time and is strongly associated with histopathologic high-risk features. To test these hypotheses, we relied on a contemporary population-based cohort of RCC patients eligible for AST after nephrectomy from the Surveillance, Epidemiology, and End Results (SEER) database (2018–2022).^13^

## Materials and Methods

### Data Source and Study Population

We identified adult patients with histologically confirmed non-metastatic RCC (International Classification of Diseases for Oncology Third Edition [ICD-O-3] site code C64.9) who underwent nephrectomy. Analyses were restricted to patients eligible for AST, defined as those who had clear-cell RCC with T3–4 (any N, any grade), N1–2 (any T, any grade), or T2 with tumor grade 4 (any N; applied only to patients with a diagnosis from 2022 onward).^1–3,6–9^ Patients with metastatic disease and no evidence of disease after metastasis-directed therapy (M1 NED) could not be reliably identified within SEER and were therefore not included in the current analyses.

The primary outcome was AST use, defined according to SEER treatment coding (item 1639, Systemic/Surgery Sequence). The study excluded patients with unknown systemic therapy status or unknown sequence of systemic therapy. The primary tumor-related variables of interest included T stage (T4 vs. T1–3), tumor grade (G4 vs. G1–3/x), and N stage (N1–2 vs. N0/x). The G4 variable was defined to include tumors with sarcomatoid features, consistent with contemporary renal cell carcinoma grading criteria.^14,15^

### Statistical Analyses

Differences between patients who received AST and those who did not were assessed using the Wilcoxon rank-sum test for continuous variables and the Pearson chi-square test for categorical variables. Multivariable logistic regression models were fitted to test whether tumor characteristics independently predict AST use, following a three-step approach. First, high-risk histopathologic features, namely, T4, G4, and N1–2, were examined individually.^16,17^ Second, the patients were stratified into intermediate-high risk (no high-risk features) versus high risk (at least one high-risk feature) groups. Third, cumulative burden was assessed by the number of high-risk features (0, 1, 2, or 3).

Covariates included tumor size (continuous), year of diagnosis (continuous), age (continuous), sex (female vs. male), race/ethnicity (Caucasian vs. non-Caucasian), marital status (married vs. unmarried), income (low [annual income below 80,000 USD] vs. high), and area of residence (urban vs. rural).^18^ These variables were included to minimize or ideally eliminate confounding related to access to care, temporal trends in treatment adoption, and baseline patient characteristics, thereby isolating the independent association between tumor-related high-risk features and AST use.

The study used R software for statistical computing and graphics (R version 4.4.1; the R Foundation for Statistical Computing, Vienna, Austria). All tests were two-sided with statistical significance set at *p* value lower than 0.05 following Bonferroni adjustment. The study was reported according to the Strengthening the Reporting of Observational Studies in Epidemiology (STROBE) statement.^19^

## Results

### Baseline Characteristics and Temporal Trend

**Fig. 1:**
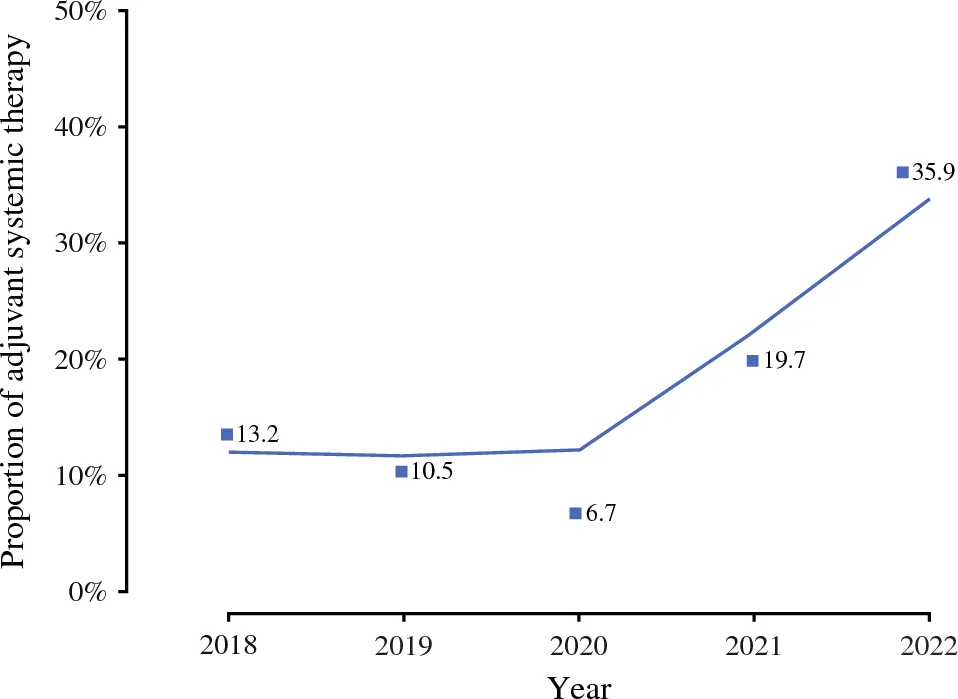
Proportion of patients receiving adjuvant systemic therapy among eligible renal cell carcinoma patients after nephrectomy over time, within the SEER database (2018–2022)

In the SEER database (2018–2022), we identified 6,497 RCC patients eligible for AST after nephrectomy (Table 1). Of these patients, 1,163 (17.9%) received AST. The annual proportion showed minor variation between 2018 and 2020 (ranging from 6.7% to 13.2%) and subsequently increased to 19.7% in 2021 and 35.9% in 2022 (*p* < 0.001; Fig. 1).

**Table 1:** Descriptive characteristics of renal cell carcinoma patients eligible for adjuvant therapy after nephrectomy, within the SEER database (2018–2022), stratified according to receipt of adjuvant systemic therapy

Characteristics	Overall (*n* = 6,497) *n* (%)	Adjuvant systemic therapy (*n* = 1,163, 17.9%) *n* (%)	No adjuvant systemic therapy (*n* =5,334, 82.1%) *n* (%)	*p* value^a^
Median year of diagnosis (IQR)	2020 (2019–2021)	2021 (2020–2022)	2020 (2019–2021)	**<0.001**
Median age: years (IQR)	65 (57–72)	63 (55–70)	65 (58–73)	**<0.001**
Male sex	4,579 (70.5)	805 (69.2)	3,774 (70.8)	0.3
Caucasian race/ethnicity	4,270 (65.7)	3,471 (65.1)	799 (68.7)	**0.018**
Married	4,205 (64.7)	3,406 (63.9)	799 (68.7)	**0.002**
Income >80,000 USD	4,116 (63.4)	3,391 (63.6)	725 (62.3)	0.4
Urban residence	5,571 (85.7)	4,587 (86.0)	984 (84.6)	0.2
Median tumor size: cm (IQR)	7.5 (5.8–9.6)	8.5 (6.5–11.0)	7.2 (5.6–9.5)	**<0.001**
T4 stage	160 (2.5)	53 (4.6)	107 (2.0)	**<0.001**
Tumor grade 4	1,530 (23.5)	423 (36.4)	1,107 (20.8)	**<0.001**
Sarcomatoid features	553 (8.5)	168 (14.4)	385 (7.2)	**<0.001**
Partial nephrectomy	559 (8.6)	64 (5.5)	495 (9.3)	**<0.001**
Lymphadenectomy performed	1,783 (27.4)	424 (36.5)	1,359 (25.5)	**<0.001**
N1–2 stage	354 (5.4)	129 (11.1)	225 (4.2)	**<0.001**
Neoadjuvant systemic therapy	127 (2.0)	50 (4.3)	77 (1.4)	**<0.001**

IQR, interquartile range; USD, U.S. dollars

^a^Wilcoxon rank sum test, Pearson’s chi-squared test

The patients receiving AST were younger (median age, 63 vs. 65 years; *p* < 0.001), less frequently Caucasian (65.1% vs. 68.7%; *p* = 0.018), and less frequently married (63.9% vs. 68.7%; *p* = 0.002). In contrast, no differences in AST use were observed for sex (*p* = 0.3), income (*p* = 0.4), or area of residence (*p* = 0.2).

### Tumor Characteristics

**Fig. 2:**
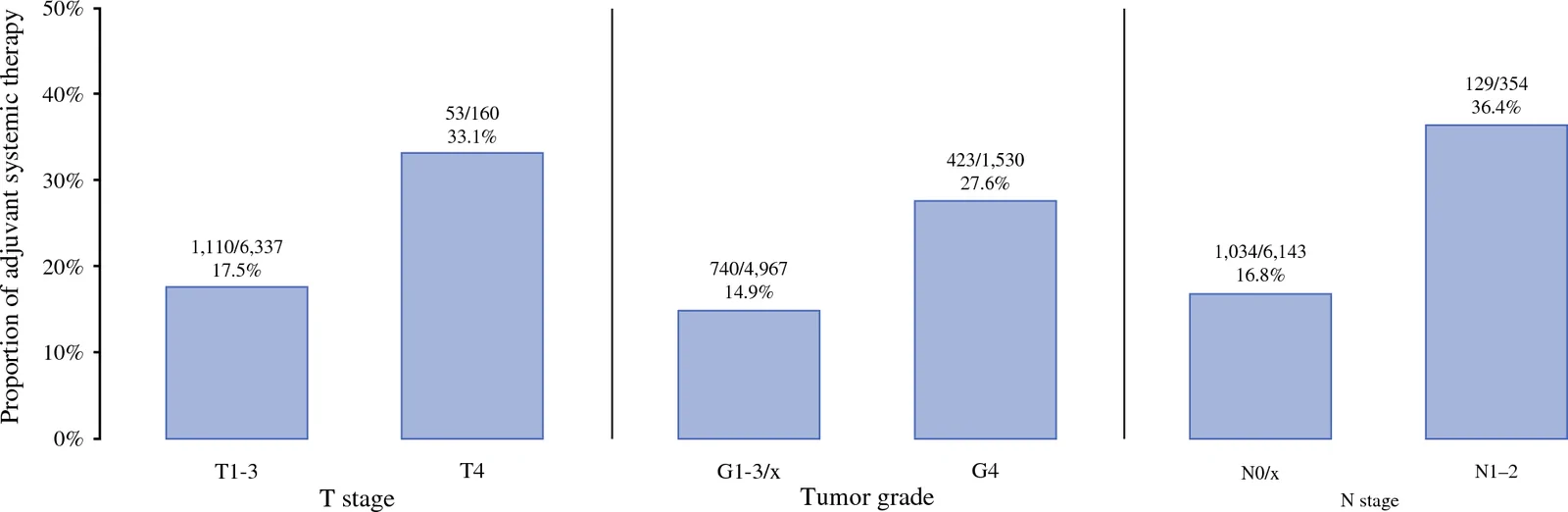
Proportion of patients receiving adjuvant systemic therapy among eligible renal cell carcinoma patients after nephrectomy, within the SEER database (2018–2022), stratified by T stage, tumor grade, and N stage

The proportions of patients receiving AST were higher among those harboring adverse tumor characteristics. Specifically, 33.1% of the patients with T4 stage received AST, compared with 17.5% of those with T1–3 stage (*p* < 0.001). The proportion of AST use also was higher among the patients with G4 tumors (27.6% vs. 14.9% for G1–3/x; *p* < 0.001). Similarly, the patients with lymph node involvement (N1–2 stage, 36.4%) had higher AST use than those without it (N0/x stage, 16.8%; *p* < 0.001, Fig. 2).

In multivariable logistic regression models, all three high-risk features were independently associated with a higher likelihood of receiving AST. Specifically, N1–2 stage (odds ratio [OR], 2.57; 95% CI, 2.00–3.29; *p* < 0.001) was the strongest predictor of AST use, followed by G4 (OR, 1.94; 95% CI, 1.67–2.25; *p* < 0.001) and T4 stage (OR, 1.68; 95% CI, 1.16–2.42; *p* = 0.006).

### Risk-Adapted Use of Adjuvant Systemic Therapy

**Fig. 3:**
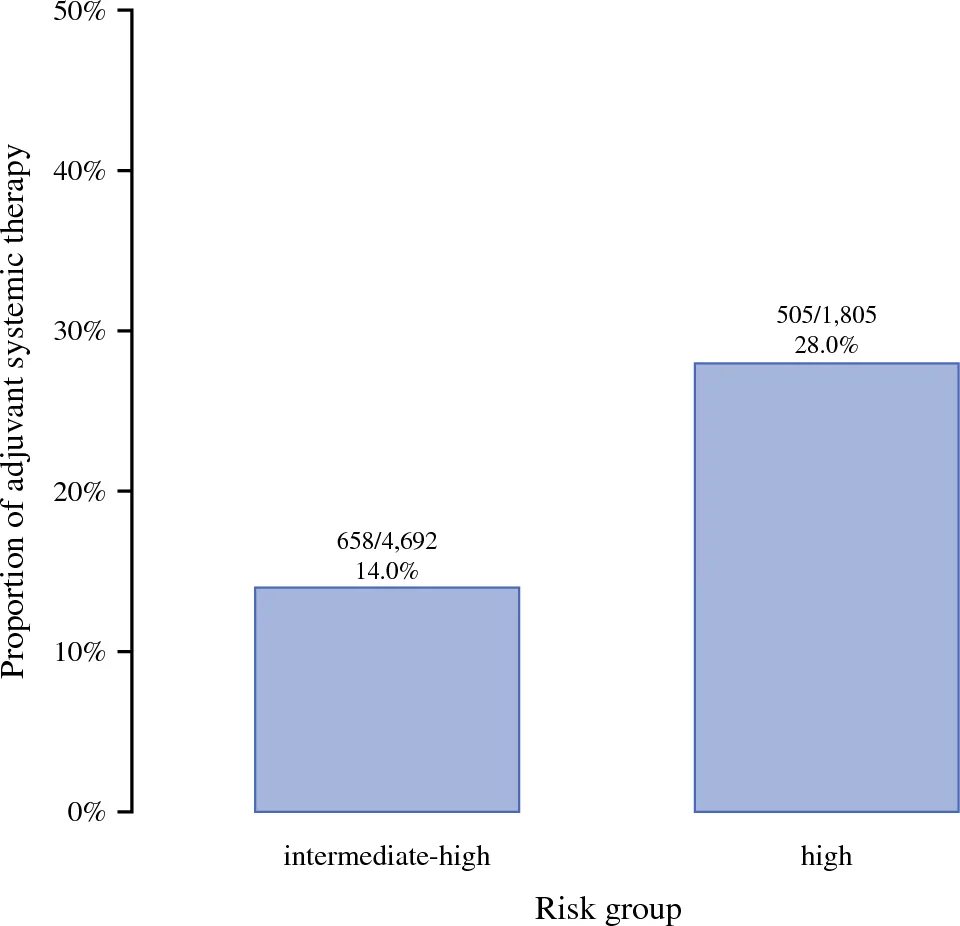
Proportion of patients receiving adjuvant systemic therapy among eligible renal cell carcinoma patients after nephrectomy, within the SEER database (2018–2022), stratified by intermediate-high-risk vs. high-risk group

Of the 6497 patients, 1805 (27.8%) harbored at least one high-risk feature (T4, G4, or N1–2) and were classified as high-risk RCC, whereas the remaining patients were classified as intermediate-high-risk RCC. Of the high-risk patients, AST was recorded for 28% compared with 14% of those with intermediate-high-risk RCC (Fig. 3). In separate multivariable logistic regression models, the presence of at least one high-risk feature independently predicted higher odds of AST use relative to the absence of high-risk features (OR, 2.27; 95% CI, 1.97–2.62; *p* < 0.001; Table 2).

**Table 2:** Multivariable logistic regression model predicting adjuvant systemic therapy among eligible renal cell carcinoma patients within the SEER database (2018–2022)

Characteristics	OR^a^	95% CI^a^	*p* value
T4 stage (ref. T1–3)	1.68	1.16–2.42	0.006
N1–2 stage (ref. N0/x)	2.57	2.00–3.29	**<0.001**
Tumor grade G4 (ref. G1–3/x)	1.94	1.67–2.25	**<0.001**
High-risk group (ref. intermediate-high)	2.27	1.97–2.62	**<0.001**
No. of high-risk features (ref. 0)			
1	2.09	1.80–2.43	**<0.001**
2	3.56	2.67–4.72	**<0.001**
3	4.04	1.26–12.15	**0.014**

OR, odds ratio; CI, confidence interval

^a^Adjusted for tumor size, year of diagnosis, age, sex, race/ethnicity, marital status, income, area of residence

**Fig. 4:**
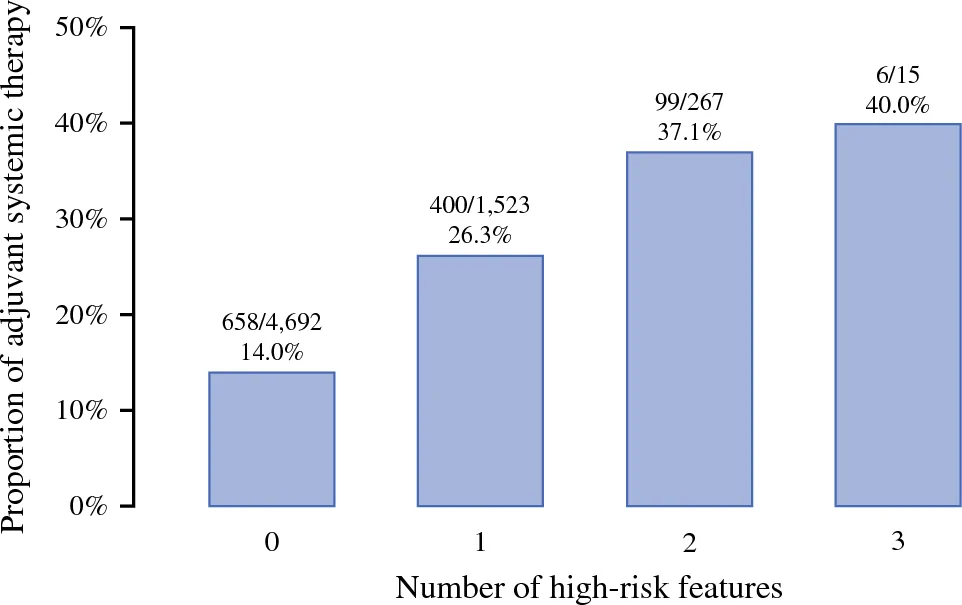
Proportion of patients receiving adjuvant systemic therapy among eligible renal cell carcinoma patients after nephrectomy, within the SEER database (2018–2022), stratified by number of high-risk features

When cumulative burden was considered, the proportion of AST use increased with the number of high-risk features. In the cohort, 4692 patients had no high-risk features (1523 had one, 267 had two, and 15 had all three high-risk features). The corresponding rates of AST use were 14.0%, 26.3%, 37.1%, and 40.0%, respectively (Fig. 4). In separate multivariable logistic regression models, an increasing number of high-risk features was independently associated with higher odds of AST use (reference: no high-risk features). Specifically, the odds ratios were 2.09 (95% CI, 1.80–2.43; *p* < 0.001) for one high-risk feature, 3.56 (95% CI, 2.67–4.72; *p* < 0.001) for two high-risk features, and 4.04 (95% CI, 1.26–12.15; *p* = 0.014) for three high-risk features (Table 2).

## Discussion

Since regulatory approvals and guideline endorsement, AST has become a standard option for selected patients after nephrectomy for RCC. However, population-based data on real-world utilization remain limited, and it is unclear whether treatment allocation aligns with established histopathologic high-risk features. We addressed these knowledge gaps using the SEER database (2018–2022) and made several noteworthy observations.

First, we relied on a large, contemporary, population-based cohort of 6497 patients. All were eligible for AST based on inclusion criteria from pivotal clinical trials, which have been subsequently adopted in current guidelines.^1–3,6–9^ To the best of our knowledge, this is the first population-based study to evaluate rates of AST use among patients eligible for AST after nephrectomy in the post-approval treatment era. Earlier analyses addressing the same endpoint were restricted to pre-approval cohorts (up to 2015), thereby capturing off-label use rather than AST administered in accordance with eligibility criteria and guideline recommendations.^10,11^ Consequently, the current study findings provide novel insight into treatment patterns in the setting of approved AST after nephrectomy for RCC.

Second, AST was administered to 18% of the RCC patients who underwent nephrectomy and harbored tumor characteristics indicating eligibility. Notably, in the first study years (2018–2020), the rate of AST use was 7% to 13%, which is higher than the 3% to 5% reported in pre-approval population-based studies (2006–2015) and may reflect early uptake after regulatory approval.^6,7^ Thereafter, AST use increased substantially, reaching 20% in 2021 and 36% in 2022. Importantly, despite this temporal trend, only a minority of eligible RCC patients received AST after nephrectomy. Ideally, a much higher proportion would be expected because offering AST would be guideline-recommended for all included patients.^14–17^ This finding indicates delays in the adoption of contemporary clinical trial evidence into routine practice of AST use and is consistent with reports from other urologic malignancies, for which AST remained underutilized despite guideline endorsement.^20–22^ However, AST utilization rates in RCC remain lower than those reported for urothelial cancer, likely because of the comparatively more favorable oncologic prognosis of localized RCC after nephrectomy.^20–22^

Third, when rates of AST use were stratified by tumor characteristics, higher rates were observed among the patients with adverse tumor characteristics. Specifically, among the patients with T4 stage, 33% received AST compared with 18% of those with T1–3 stage. Among the patients with G4 tumors, 28% received AST compared with 15% of those with G1–3/x tumors. Among the patients with N1–2 stage, 36% received AST compared with 17% of those with N0/x stage. In multivariable analyses, all three high-risk features remained independent predictors of AST use, with N1–2 stage disease representing the strongest predictor (OR, 2.57), followed by G4 (OR, 1.94) and T4 stage (OR, 1.68). Importantly, all multivariable analyses were adjusted for year of diagnosis and sociodemographic characteristics to account for the previously described temporal increase in AST and potential differences in access to care. These findings indicate that in contemporary real-world practice, AST use reflects established pathologic risk factors incorporated into guideline recommendations.^1–3,18,23,24^ This observation is consistent with prior studies from the pre-approval era, which similarly reported preferential use of AST for RCC patients with more aggressive tumor characteristics.^10,11^

Fourth, combined assessment of the three histopathologic high-risk features enabled a more refined risk stratification. Specifically, the patients with at least one high-risk feature (classified as high-risk RCC) had a 2.3-fold higher likelihood of receiving AST (28% vs. 14%) than those without high-risk features (classified as intermediate-high-risk RCC).

To further explore potential cumulative effects, the association between the number of high-risk features and AST use was examined. A stepwise increase in rates of AST use with increasing number of high-risk features was observed. Specifically, AST use was recorded for 26% of the patients with one high-risk feature, in 37% of those with two high-risk features, and in 40% of those with all three high-risk features. This gradient remained consistent in multivariable analyses, with the magnitude of the adjusted associations closely mirroring the univariable rates of AST use (OR, 2.09, 3.56, and 4.04, respectively). These findings demonstrate a cumulative effect of the three high-risk features, resembling a dose-response relationship. Such patterns suggest that real-world use of AST may be driven by perceived recurrence risk, in line with risk stratification approaches derived from clinical trials and subsequent consensus and guideline recommendations.^1–3,6–9,17^.

An additional consideration relates to histologic subtype. Contemporary evidence supporting AST is strongest for clear-cell RCC, which represented the population evaluated in the pivotal AST trials and consequently forms the basis of current guideline recommendations.^1–3,6–9,17^ Accordingly, the current study was intentionally restricted to patients with clear-cell RCC. Therefore, the current findings cannot be generalized to non–clear-cell RCC or variant histologies, for which evidence supporting AST remains limited, and treatment decisions are frequently individualized.^25^

From a clinical perspective, the observed overall rates of AST use appear suboptimal with respect to adoption of novel guideline-endorsed cancer treatments. However, an increase in AST use was recorded during the study period, predominantly in the final 2 years of observation. This temporal trend is more encouraging than the overall utilization rates alone suggest. In addition, the observed risk-adapted pattern of use may be appropriate because RCC patients with higher risk of recurrence are more likely to benefit from AST after nephrectomy. Nevertheless, the observation that only 40% of patients harboring all three high-risk features (T4, G4, and N1–2) received AST indicates potential underutilization among those at highest risk of recurrence. This finding should be interpreted with caution because the subgroup comprising all three high-risk features was relatively small, even within this large population-based cohort, resulting in limited precision of the corresponding effect estimate.

Furthermore, it should be acknowledged that not all eligible patients are suitable candidates for AST. Treatment decisions are based on shared decision-making and patient preferences, whereas contraindications to tyrosine kinase inhibitors or immune checkpoint inhibitors, comorbidity burden, and limited life expectancy may further preclude treatment for a subset of patients. Moreover, the availability of effective salvage treatment options in the event of recurrence may further reduce the perceived need for immediate postoperative systemic treatment. Contemporary management of recurrent RCC increasingly relies on active surveillance of selected patients, kidney-sparing treatment for selected local recurrences, metastasis-directed therapies, and highly effective systemic treatments.^1–3,17,26^

Current guidelines recommend offering rather than mandating AST, emphasizing shared decision-making that incorporates patient preferences, including concerns regarding toxicity and potential overtreatment. Consequently, treatment rates approaching 100% are neither expected nor necessarily appropriate in routine clinical practice, particularly given ongoing debate regarding the optimal role of AST in RCC and the evolving evidence base supporting its use.^4,5^

Several limitations of this study warrant acknowledgment. First, the current analysis was subject to the inherent constraints of retrospective analyses based on registry data.^10,11,13,27^ In particular, SEER does not provide detailed information on the type of systemic therapy administered, thereby precluding differentiation between specific agents such as tyrosine kinase inhibitors and immune checkpoint inhibitors. Second, comorbidity burden and insurance status are not captured in current releases of SEER, which may result in residual confounding despite multivariable adjustment.^28–30^ Finally, prospective trials of adjuvant tyrosine kinase inhibitors have not demonstrated a consistent overall survival benefit, whereas more recent evidence supporting adjuvant immunotherapy is emerging.^9^ Consequently, the increase in AST utilization observed during the later study years may largely reflect the adoption of adjuvant pembrolizumab rather than a generalized increase in the use of all AST modalities. Moreover, evaluation of real-world oncologic outcomes after AST will require more mature follow-up evaluation than is currently available in SEER. In particular, the limited number of cancer-specific mortality events after the introduction of adjuvant pembrolizumab and the absence of disease- or metastasis-free survival data currently preclude meaningful population-based effectiveness analyses. Therefore, future studies should assess treatment patterns as well as oncologic outcomes in the era of adjuvant immune checkpoint inhibitors as more mature data become available.

Taken together, although the proportion of AST use increased over time, only a minority of eligible RCC patients received AST after nephrectomy. Use of AST was strongly associated with adverse tumor characteristics and increased in a stepwise fashion with the accumulation of high-risk features. Nevertheless, AST use remained limited even among patients at the highest risk, namely, those with T4 G4 N1–2 RCC.

These findings suggest potential underutilization of AST among those most likely to benefit. Targeted efforts within the urology and oncology communities to enhance awareness, implement guideline recommendations, and encourage multidisciplinary decision-making may help optimize the use of AST in routine clinical practice.

## Data Availability

The data in this study are derived from the Surveillance, Epidemiology, and End Results (SEER) Program database of the National Cancer Institute. Access to SEER data is available upon request through the SEER*Stat software after registration and approval by the National Cancer Institute. The authors had authorized access to these data. The data are not publicly available due to privacy and confidentiality restrictions imposed by the SEER program.
